# Shape optimization design of the offset mirror in FEL-1 beamline at S^3^FEL

**DOI:** 10.1038/s41598-023-36645-9

**Published:** 2023-06-14

**Authors:** Zhongmin Xu, Weiqing Zhang, Chuan Yang, Yinpeng Zhong

**Affiliations:** 1 Institute of Advanced Science Facilities, Shenzhen (IASF), No. 268 Zhenyuan Road, Guangming District, Shenzhen, 518107 People’s Republic of China; 2 Dalian Institute of Chemical Physics, Chinese Academy of Sciences, Dalian, 116023 People’s Republic of China

**Keywords:** Mechanical engineering, Applied optics, Optical techniques

## Abstract

Nowadays, due to the advantages of high peak power, high average power, ultra-short pulse, and fully coherent characteristics, the high-repetition-rate free-electron laser (FEL) is thriving in many countries around the world. The thermal load caused by high-repetition-rate FEL poses a great challenge to the mirror surface shape. Especially in the case of high average power, how to perfectly control the mirror shape to maintain the coherence of the beam has become a difficult problem in beamline design. In addition to multi-segment PZT, when multiple resistive heaters are used to compensate for the mirror shape, the heat flux (or power) generated by each heater must be optimized to obtain sub-nanometer height error. This article establishes MHCKF model for the mirror surface deformation under the combined effect of the mirror initial deformation, the thermal deformation caused by X-rays, and the deformation compensated by multiple heaters. By searching the perturbation term in the mathematical model, the least squares solution of the heat fluxes generated by all heaters can be obtained. This method can not only set multiple constraints on the heat fluxes but also quickly obtain their values when minimizing the mirror shape error. It overcomes the problem of time-consuming optimization processes encountered by traditional finite element analysis software, especially in the context of multi-parameter optimization. This article focuses on the offset mirror in the FEL-1 beamline at S^3^FEL. Using this method, the optimization of 25 heat fluxes generated by all resistive heaters was accomplished within a few seconds utilizing an ordinary laptop. The results indicate that the height error RMS decreased from 40 nm to 0.009 nm, and the slope error RMS reduced from 192.7nrad to 0.4nrad. Wave-optics simulations show that the wavefront quality has been significantly improved. In addition, some factors affecting mirror shape error, such as the number of heaters, higher repetition rate, film coefficient, and the length of copper tube, were analyzed. The results show that the MHCKF model and optimization algorithm can effectively solve the optimization problem of compensating for the mirror shape with multiple heaters.

## Introduction

**Figure 1:**
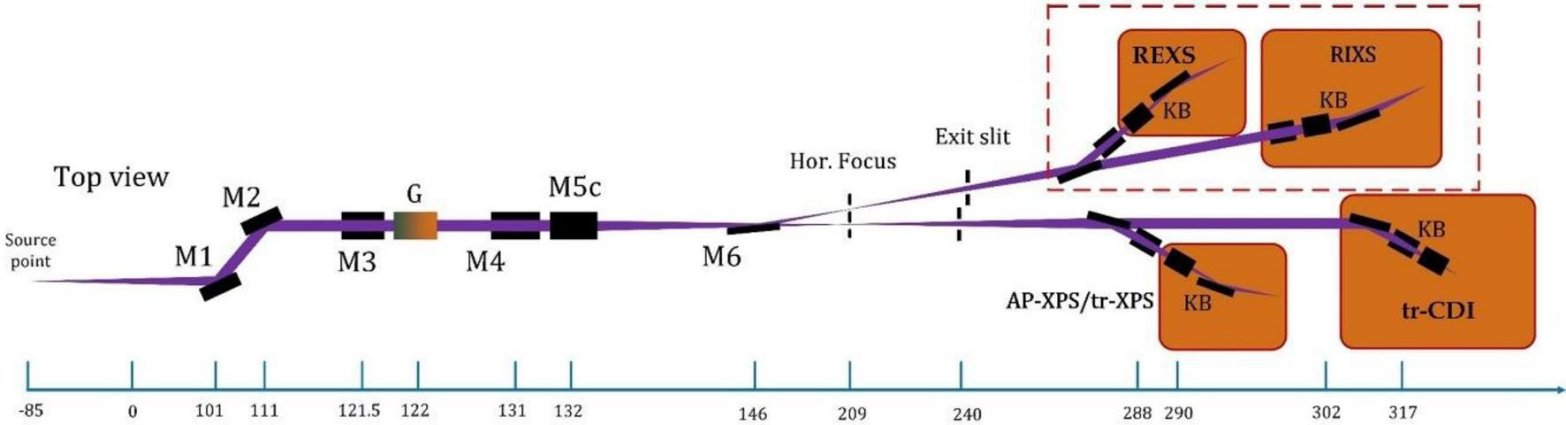
Optical layout of FEL-1 beamline at S^3^FEL.

In recent years, with the rapid development of superconducting technology, it is possible to develop high-repetition-rate X-ray free electron laser (FEL) user facility. There are several facilities under design or construction around the world, such as the European X-ray free-electron laser (XFEL)^1^, FLASH^2^, the Linac Coherent Light Source II (LCLS-II)^3^, and Shanghai High-repetition Hard X-ray Free Electron Laser (SHINE)^4^. In China, in addition to SHINE, the Shenzhen Superconducting Soft X-ray Free Electron Laser (S^3^FEL) is a new light source under the proposal phase at the Institute of Advanced Science Facilities (IASF), Shenzhen. S^3^FEL consists of a 2.5 GeV CW superconducting linear accelerator and four initial undulator lines, aiming to generate X-rays between 40 eV and 1.24 keV at rates up to 1 MHz^5^. The first phase of S^3^FEL includes four beamlines, among which FEL-1 will operate in the SASE mode with a repetition rate of up to 100 kHz. The optical layout of FEL-1 is shown in Fig. 1.

The FEL-1 beamline aims to construct three experimental endstations, including the time-resolved coherent diffraction imaging station (tr-CDI), surface ambient-pressure X-ray photoelectron station (AP-XPS), and resonant soft X-ray scattering station (including RIXS and REXS), respectively. As shown in Fig. 1, many mirrors are used to meet the requirements of experimental endstations. The first mirror in the beamline, the offset mirror (M1), is critical in maintaining photon and wavelength stability. According to the Maréchal Criteria^6^, for coherent transmission, the height error RMS of the offset mirror should be less than 0.9 nm and the slope error RMS should be less than 100nrad, which are more stringent than those of the mirrors in synchrotron radiation facilities. Therefore, it is necessary to choose an appropriate shape control scheme.

So far, many shape control schemes have been used for mirrors in synchrotron radiation and FEL facilities, including passive and active methods. At Advanced Photon Source (APS), a contact-cooled method, in which two water-cooled blocks are clamped against the two side surface of a mirror, is used in an X-ray undulator beamline^7^. Xu proposed a Local-Side-Cooling scheme^8^ to lower the slope error for a collimation mirror at Shanghai Synchrotron Radiation Facility (SSRF), while Zhang used water cooling for a mirror with notches^9^ at European Synchrotron Radiation Facility (ESRF). All of these cases are passive. Although they can reduce the slope error under certain boundary conditions (such as a single incident wavelength), it is not possible to obtain the surface shape that satisfies the entire wavelength range requirement. Therefore, active shape control schemes have been more widely used. At ESRF, Signorato used multi-segmented piezoelectric to correct the shape of the mirror^10^. Bimorph mirrors based on PZT have been used on several beamlines at Diamond Light Source (DLS)^11–13^ as well. Yang used multiple PZT elements to control the shape of the mirror at the XFEL^14^, and Zhang proposed the REAL method^15^, which uses several resistive heaters to compensate for the shape of the mirror at SLAC. A prototype was also developed and tested on the LCLS-II^16^. In both of these active control methods, the bimorph mirror has a stronger ability to correct the shape because it uses more piezoelectric elements. However, it also brings many problems, such as PZT bonding, high voltage control, and curvature drift^17^. By comparison, for the latter scheme, the mirror and the resistive heaters are separate and no high voltage control is required, making it easier to implement and having better shape stability.

These active control schemes provide a great degree of freedom to adjust the shape of the mirror, reducing the shape error significantly compared with traditional schemes. However, to achieve the optimal mirror shape, how to find the values for so many parameters becomes a challenge. It would be a very time-consuming task to directly use finite element analysis (FEA) software to optimize the design of multiple parameters. In the worst case, it may not be possible to find a suitable solution. Moreover, this does not apply to online real-time adaptive control of the mirror shape. So far, there have been many algorithms for analyzing multiple PZT voltages^18–20^. However, the piezoelectric effect of PZT and the thermal effect of the resistive heater are different in terms of adjusting the mirror shape. For the former, voltages in different directions will produce deformation in different directions, whereas for the heater, it is not the case. This paper establishes a perturbation model for the mirror surface shape deformation under the combined effect of initial mirror deformation, X-ray-induced thermal deformation, and deformation compensation by multiple heaters, and proposes a shape optimization algorithm to solve the problem of surface shape compensation using resistive heaters.

### Mirror model and boundary condition

**Figure 2:**
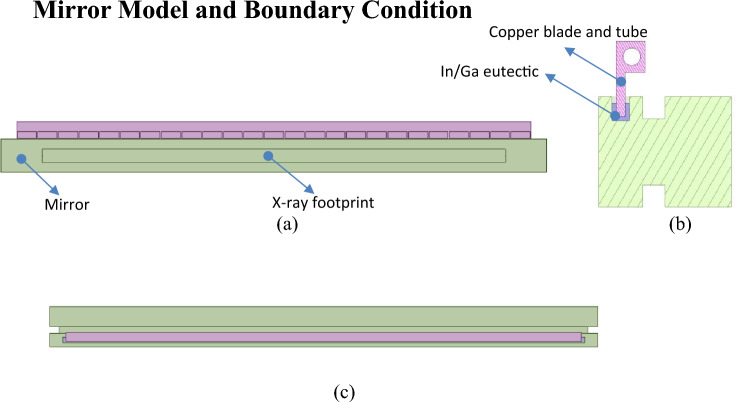
Different views of FEL-1 offset mirror and shape compensation system. (**a**) Front view ;(**b**) Enlarged section view; (**c**) Top view.

This paper establishes a 3D model and shape compensation system for the FEL-1 offset mirror in Ansys Workbench, as shown in Fig. 2, similar to the prototype at SLAC. As a plane mirror (green part), its specifications are listed in Table 1. In the front view, the black rectangle on M1 represents the X-ray footprint. A trough is opened above the mirror, filled with a liquid In/Ga eutectic (blue cross-section line in Fig. 2b). The cooling of the system is achieved by partially immersing the copper blade into In/Ga eutectic, while the copper tube are used to circulate cooling water. Its inner diameter of the tube is 8 mm, and the applied heat transfer coefficient is 3E-3 W/mm^2^/°C. 25 resistive heaters are attached to the copper blade for compensating the mirror shape. To simplify the model, all heaters have been omitted. Instead, 25 rectangles representing the positions of the heaters are drawn on the copper blade, with each rectangle measuring 30 mm*10 mm, and a distance of 2 mm between two rectangles. The corresponding heat flux generated by each heater is applied equivalently on the rectangle. Table 2 lists the corresponding material properties for finite element analysis.

**Table 1:** Specifications for the offset mirror (M1).

Substrate	Silicon
Coating	B_4_C
size (${\mathrm{mm}}^{3}$)	850 × 50 × 60
Effective size (${\mathrm{mm}}^{2}$)	800 × 30
Shape	Plane
Grazing angle for 1 nm X-ray $(\mathrm{mrad})$	7

**Table 2:** Material properties for finite element analysis.

Material	Density (kg/m^3^)	Thermal conductivity (W/m/°C)	Thermal expansion coefficient (10^–6^/°C)	Young’s modulus (GPa)	Poisson’s ratio
Silicon	2329	148	2.5	112.4	0.28
OFHC	8900	391	17.5	110	0.34
In/Ga	6350	28			

**Figure 3:**
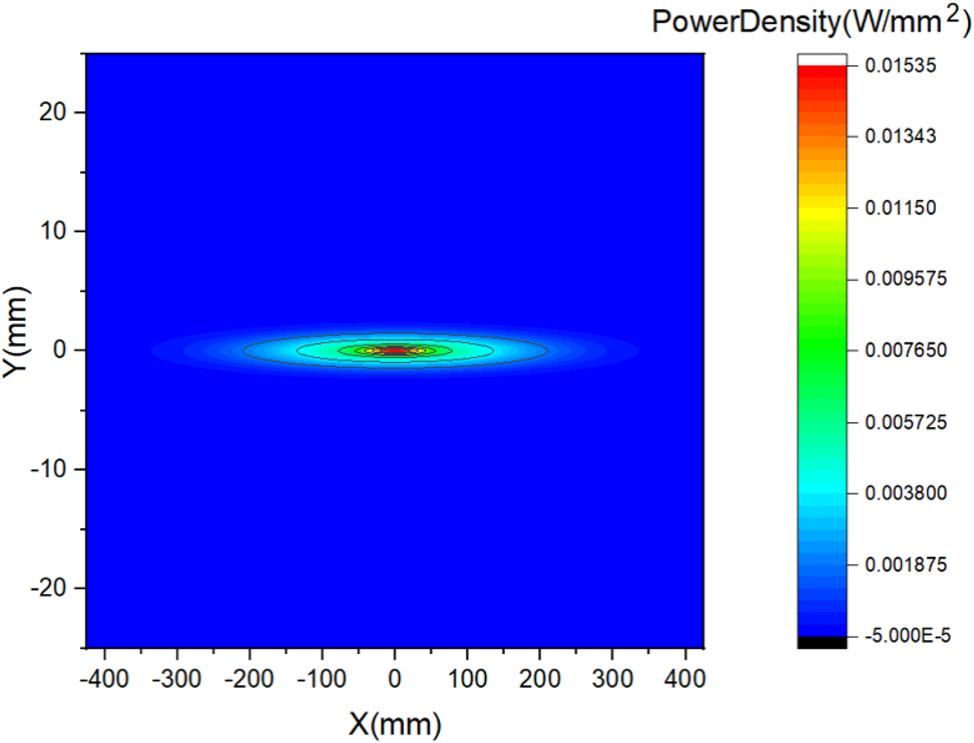
Power density distribution of wavelength 1 nm.

For X-rays with a wavelength of 1-3 nm, the absorption of M1 is less than 10%, and the total average absorbed power is below 10W. In this paper, the power density distribution of wavelength 1 nm is considered, as shown in Fig. 3. In this case, including the power of higher harmonics, the total power absorbed by M1 is 5.4W. In addition, the contact thermal conductance between the copper blade, In/Ga eutectic, and the mirror is set to 0.15W/mm^2^/°C^21^.

To evaluate the mirror surface shape, the height error and slope error RMS of the centerline in the meridional direction is usually used as the criterion. The smaller the value, the better the surface shape. In this case, it is necessary to evaluate the surface shape in the meridional direction from − 360 mm to 360 mm.

### Mathematical model

The surface shape compensation by the heaters is to apply heat flux to compensate the mirror initial deformation and X-ray-induced thermal deformation, so as to minimize the error between the actual deformation and the ideal deformation. Based on this idea, a mathematical model called the MHCKF model was established, as shown in Eq. (1)

1 $$M\left(x\right)H+C\left(x\right)+K\left(x\right)=F\left(x\right)$$

where *M(*$x$*)* is the response function of the resistive heaters, which is a matrix, *M(x)*$\in {R}^{m\times n}$. m is the number of sampling points on the centerline of the footprint. And n is the number of the resistive heaters; *H* is a series of the heat fluxes generated by the heaters and is a column vector, *H* $\in {R}^{n}$. The first term, $M\left(x\right)H$, represents the compensating deformation caused by many resistive heaters; *C(x)* is the mirror initial deformation caused by the processing, clamping, and gravity, etc. It represents the initial condition and can be used as background, *C(x)* $\in {R}^{m}$; *K(x)* is the deformation in the meridional direction caused by the X-ray power, which is also a column vector, *K(x)* $\in {R}^{m}$; *F(x)* represents the actual deformation generated by the three left terms, *F(x)*$\in {R}^{m}$.

In our case, it was found that the ideal shape for *F(x)* is a straight line (in fact, a perfectly flat surface), and its intercept value in the Cartesian coordinate system is close to the maximum thermal deformation caused by X-rays. Therefore, *F(x)* can be written as follows.

2 $$F\left(x\right)\approx (max\left(K (x)\right)+\varepsilon )I$$

where $max\left(K (x)\right)$ is the maximum deformation value of *K(x)*; $\varepsilon$ is a perturbation term, which is a scalar; *I* is a column vector of all 1 s, *I* $\in {R}^{m}$ .

From the expression Eqs. (1) and (2) you can get expression Eq. (3).

3 $$M\left(\mathrm{x}\right)H+C\left(x\right)+K\left(x\right)\approx \left(max\left(K (x)\right)+\varepsilon \right)I$$

The least squares solution of *H* can be calculated using expression Eq. (4).

4 $$H\approx {\left({M}^{T}\left(\mathrm{x}\right)M\left(\mathrm{x}\right)\right)}^{-1}{M}^{T}\left(\mathrm{x}\right)(-C\left(x\right)-K\left(x\right)+\left(max\left(K (x)\right)+\varepsilon \right)I)$$

where *T* represents the transposed matrix; − 1 stands for inverse matrix.

The residual shape error can be evaluated using the expression Eq. (5)

5 $$e=M\left(\mathrm{x}\right)H+C\left(x\right)+K\left(x\right)-(\left(max\left(K (x)\right)+\varepsilon \right)I)$$

where e is a column vector, through which the height error and slope error can be calculated.

### FEA results before shape compensation

In the absence of shape compensation, the boundary conditions only include the X-ray power absorbed by the mirror and the convection applied to the inner wall of the copper tube.

**Figure 4:**
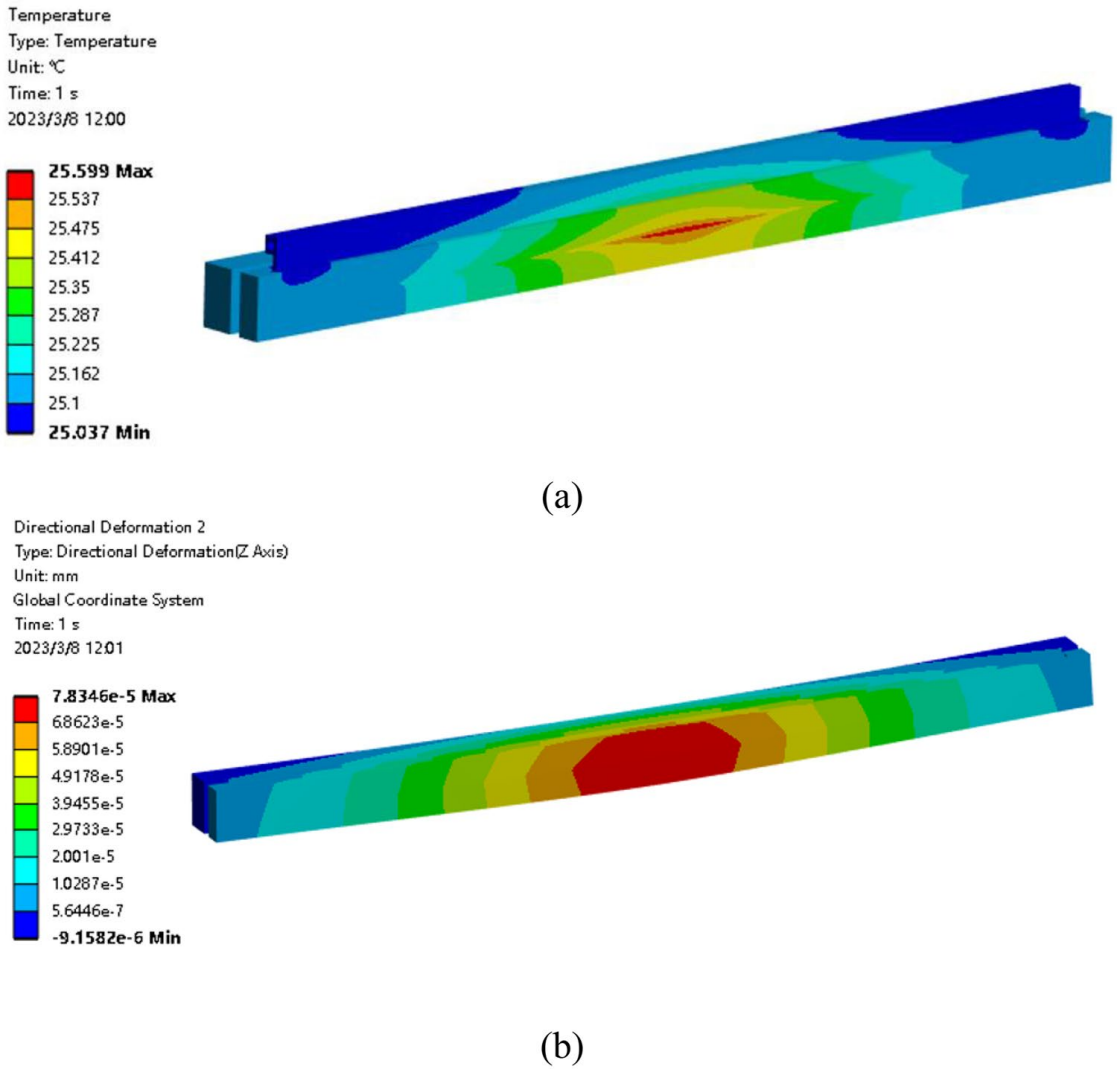
FEA results before shape compensation. (**a**) Temperature distribution; (**b**) Directional Deformation distribution.

As shown in Fig. 4, the high temperature and large deformation areas inside the mirror surface are concentrated in a small range. Therefore, there is a large temperature and deformation gradient on the centerline in the meridional direction.

**Figure 5:**
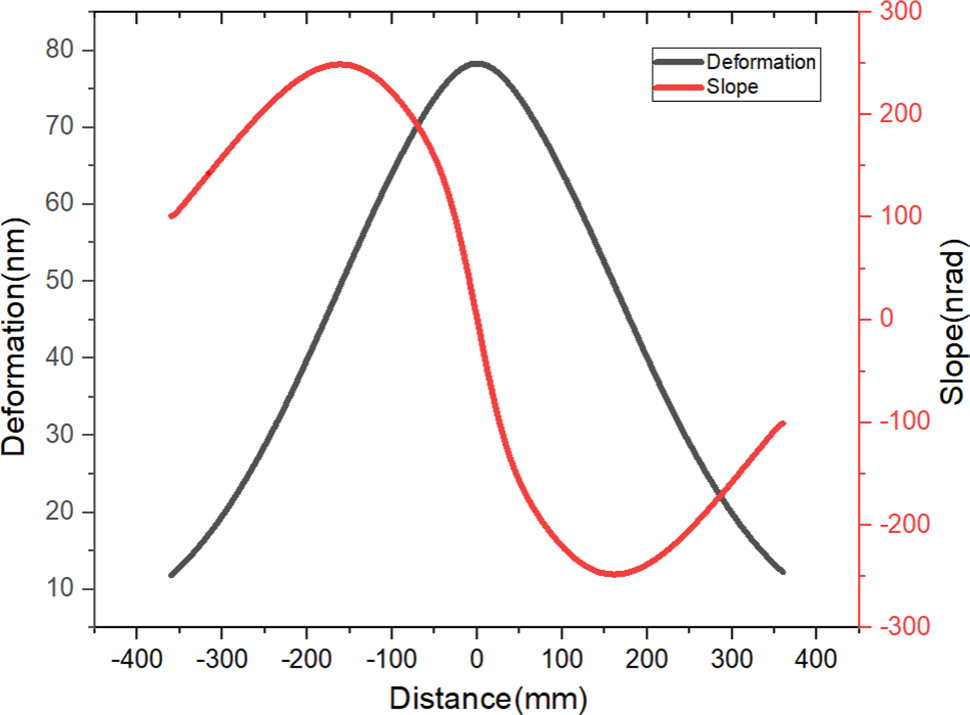
Deformation and slope curve of the centerline.

As shown in Fig. 5, although the mirror only absorbs 5.4 W of thermal power, the height error PV has reached 66.4 nm, and the RMS is 40 nm. The slope error PV is 496.9nrad, and the RMS is 192.7nrad. All of these data greatly exceed the requirements. Therefore, to obtain a high-precision mirror shape, an ordinary cooling scheme is ineffective. And a shape compensation scheme is necessary.

### Optimization algorithms and procedure

**Figure 6:**
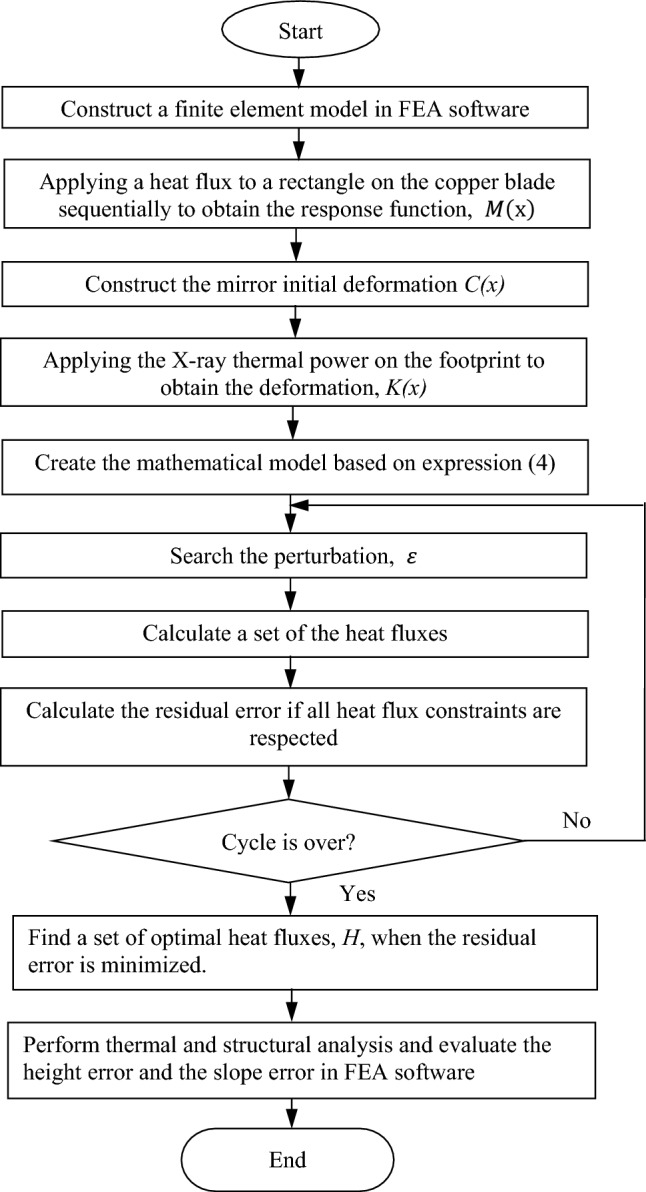
Flow chart of the optimization algorithm for searching the optimal *H*.

If there is no prototype, and it is still in the simulation and analysis phase, a flowchart of optimization algorithms for compensating the mirror shape is proposed as show in Fig. 6, based on the above MHCFK model. First, a finite element model of the entire system including the mirror, In/Ga eutectic, the copper tube and blade is created in FEA software. Second, to calculate the response function of each resistive heater, a certain heat flux value should be applied to each rectangle on the copper tube one by one, and the deformation of the mirror can be calculated. Then, the response function *M(x)* can be obtained. Next, the mirror initial deformation *C(x)* should be constructed. The influence of gravity and clamping on deformation should be obtained through structural static analysis by FEA software. As for the surface shape of the processed mirror, it can be virtually assumed. Then, the X-ray thermal power is applied on the footprint to obtain the normal deformation *K(x)*. After that, a mathematical model can be constructed based on the expression Eq. (4). Then, the perturbation term, ε, can be searched in a loop, and the heat fluxes generated by all heaters are calculated.

For a piezoelectric mirror, a voltage applied to each PZT can be positive or negative values. However, in our case, the heat fluxes generated by the heaters may not be negative, which can be set as constraints. In different applications, additional constraints can be added if there are additional requirements for heat flux. If the heat flux meets the constraint conditions, the residual shape error (height error or slope error) can be calculated. After the entire loop, a set of optimal heat fluxes can be found when the residual shape error is minimized. Finally, all heat fluxes are inputted to FEA software to evaluate the thermal, deformation, and surface shape results.

If the prototype is built, from the right-hand side of the expression Eq. (4), it can be seen that only $\varepsilon$ is unknown, and other parameters can be obtained through measurement. Therefore, by setting constraints on *H* and continuously searching for $\varepsilon$, the optimal heat fluxes can be found to achieve the minimum surface shape error.

In this paper, since the mirror is placed as shown in Fig. 2, the normal deformation of the mirror surface is minimally affected by gravity. In addition, for such high-precision mirrors, the surface shape error caused by processing is also very small. If an appropriate clamping scheme is also used, the total deformation of the mirror can be controlled within a very low range. Therefore, *C(x)* is not considered in the simulation of this paper.

### Heaters response functions (HRF)

**Figure 7:**
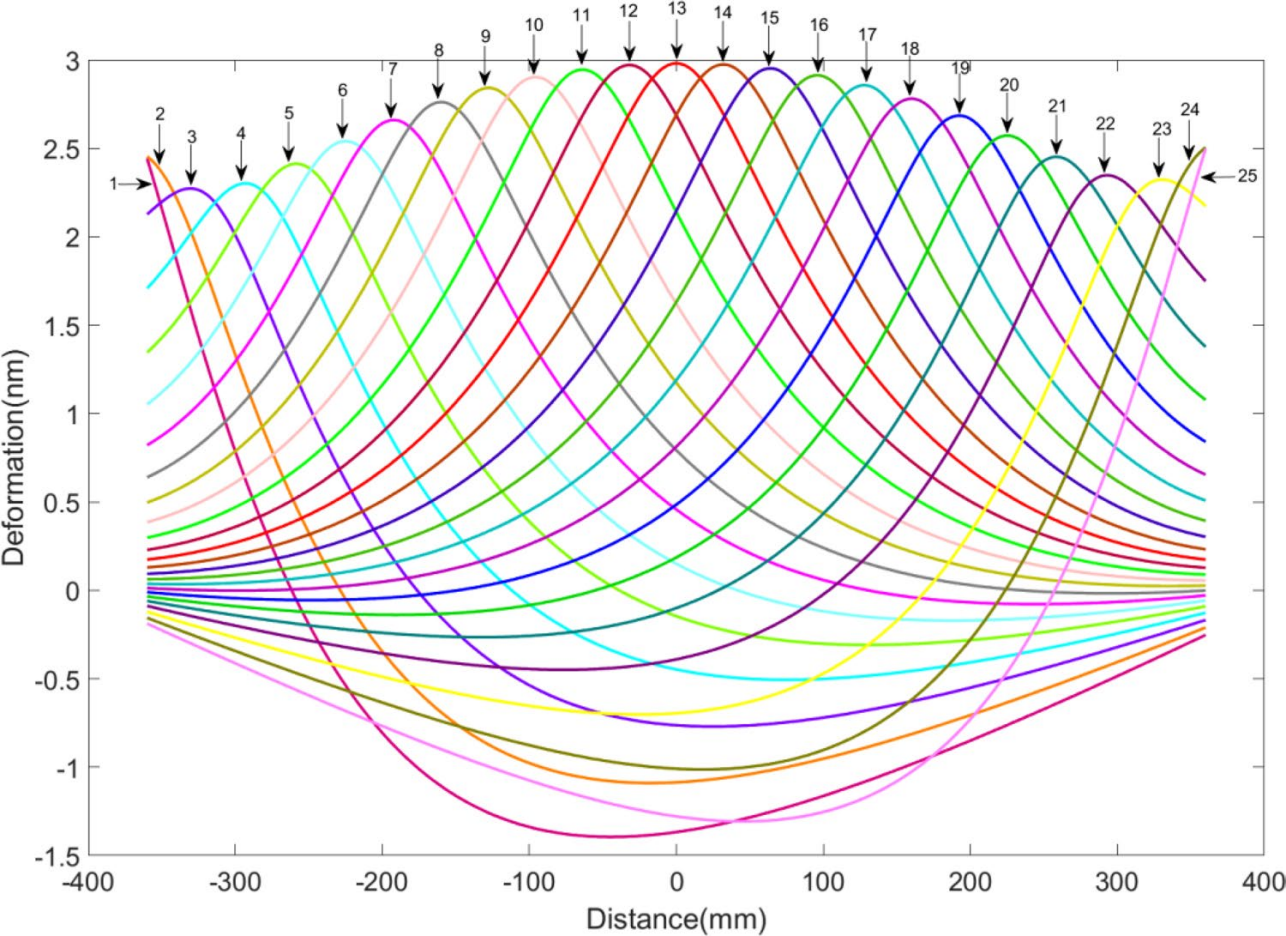
The deformation curves calculated in Ansys sequentially.

To obtain the response function of each heater shown in Fig. 2, apply a heat flux of 0.001W/mm^2^ sequentially to each rectangle on the blade using Ansys Workbench. After thermal analysis, all deformation curves [See Supplementary Information for relevant data] are shown in Fig. 7. Finally, these deformations values are divided by 0.001W/mm^2^ to obtain the Heaters Response Functions (HRF) of the heaters.

**Figure 8:**
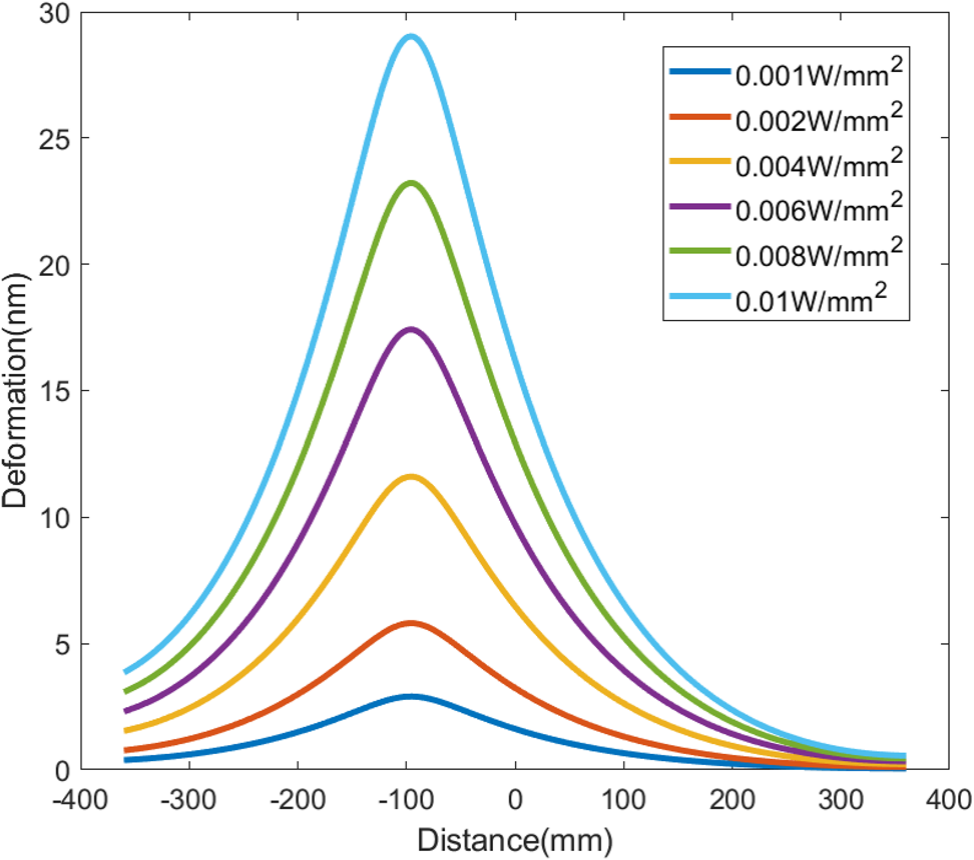
Deformation induced by different heat fluxes generated by the tenth heater.

**Figure 9:**
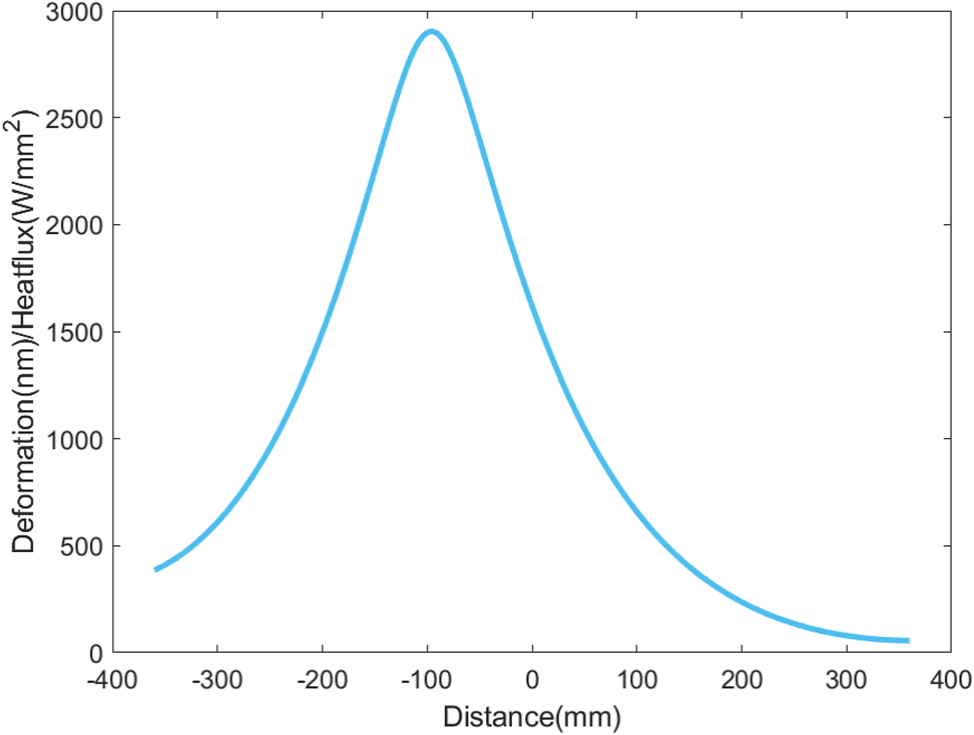
Response function for different heat fluxes for the tenth heater.

To evaluate the linearity of the response function for each heater, the 10th heater was randomly selected as the test object. Heat fluxes of 0.001 W/mm^2^, 0.002 W/mm^2^, 0.004 W/mm^2^, 0.006 W/mm^2^, 0.008 W/mm^2^, and 0.01 W/mm^2^ were sequentially applied, using Ansys to analyze the deformation of the centerline in the meridional direction. The deformation curves are shown in Fig. 8. Then, each deformation data was divided by the corresponding heat flux to obtain the response function for unit heat flux, as shown in Fig. 9. It can be seen that the six response function curves coincide perfectly, indicating the good linearity of the 10th heater response.

Usually, multi-parameter optimization design in FEA software needs optimization tools. For Ansys workbench, in addition to its own DesignXplorer, it also supports various third-party optimization tools, such as GENESIS, iSIGHT, OptiSlang, etc. When they are used for optimization design, it is necessary to select optimization variables, set upper and lower limits, choose the objective function, and then select a suitable optimization algorithm, such as genetic algorithm, particle swarm optimization, simulated annealing, etc. Whenever a new set of values is assigned to the optimization variables, the entire finite element model needs to be recalculated. If there are many variables, the computational cost will be exponential. Therefore, this type of optimization design must be carried out using high-performance computing servers.

The system as shown in Fig. 2, can be considered as a linear system. According to the MHCKF model and optimization algorithm above-mentioned, the optimization of heat fluxes generated by all heaters can be performed without FEA software. This not only eliminates the need for optimization tools, but also saves a lot of calculation workload. The optimization of heat fluxes only requires simple matrix operations using ordinary tools (NumPy or MATLAB). This method not only allows multiple constraints to be set on the heat fluxes generated by the heater but also can be completed quickly within seconds using an ordinary laptop. This solves the problem of time-consuming optimization by the traditional FEA software.

## Optimization results and discussion

**Figure 10:**
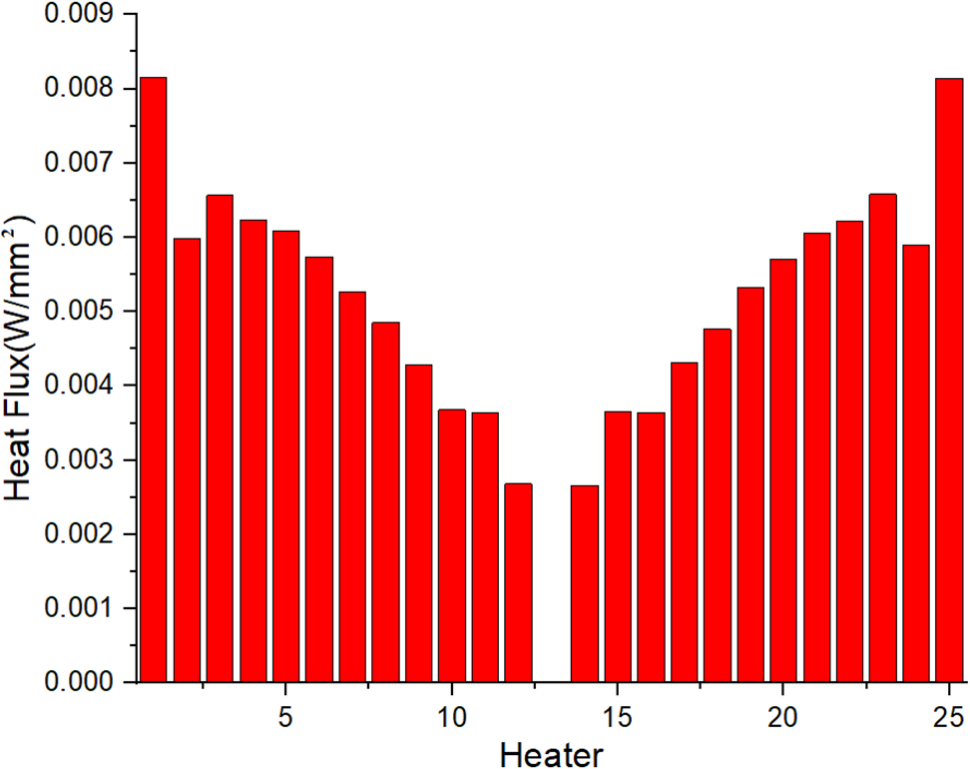
Heat Fluxes through optimization.

**Figure 11:**
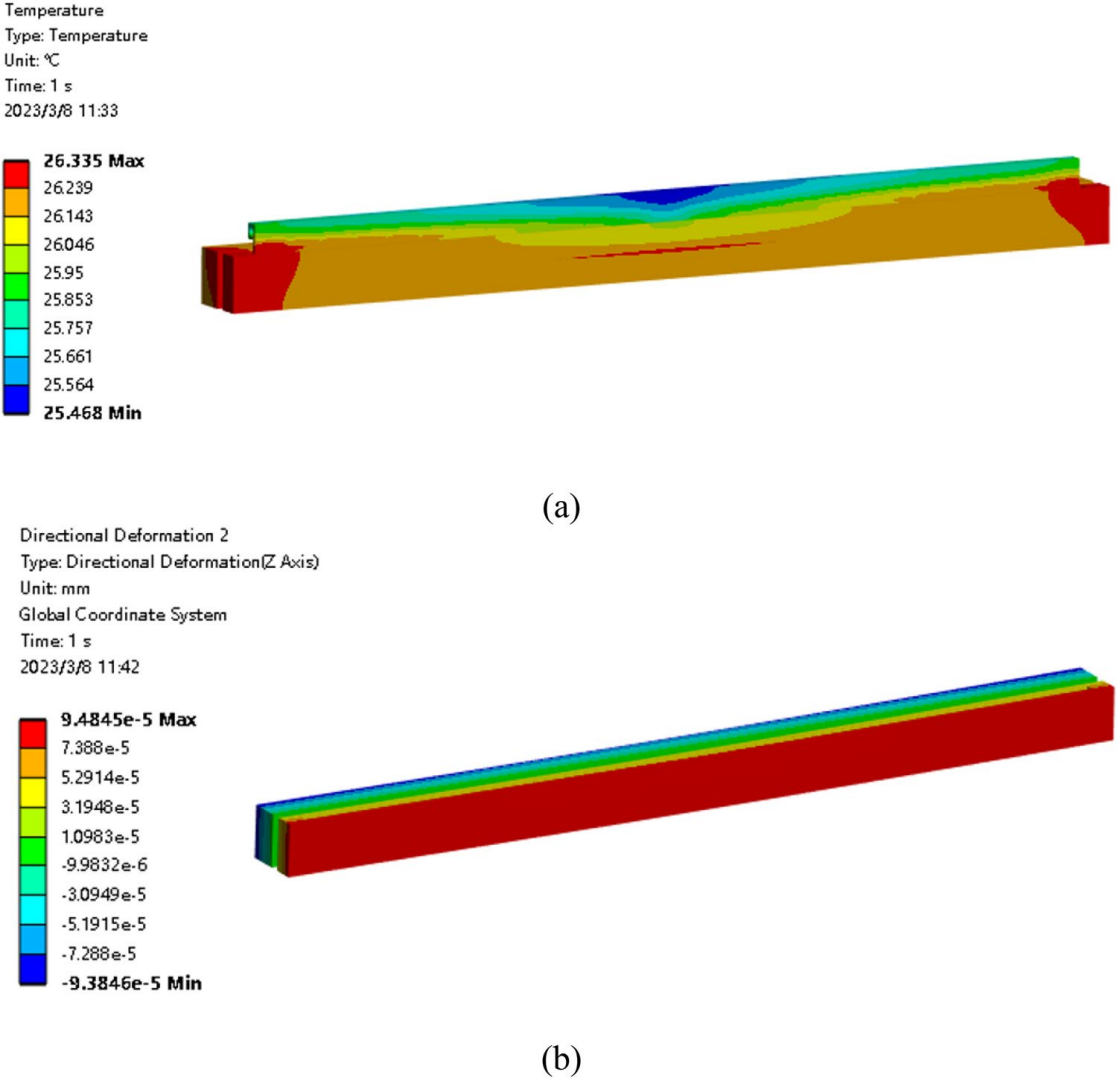
FEA results after shape compensation. (**a**)Temperature distribution; (**b**) Directional Deformation distribution.

The heat fluxes [See Supplementary Information for relevant data] are calculated through optimization, as shown in Fig. 10. And the perturbation value ε is 1.638E − 5 mm, which is of the same order of magnitude as the maximum deformation (7.833E − 5 mm) when only the X-ray thermal load and water convection are applied. Then, these data are inputted into Ansys to analyze the temperature, deformation, and shape errors, as shown in Fig. 11. It can be seen that the temperature and deformation gradients in the meridional direction are very small. In this case, the total power from the 25 resistive heaters is 37.8 W. Due to the relatively low maximum temperature of the system, all radiation was not considered in the simulation. Although the maximum temperature of the mirror increases by 0.736 °C compared with the temperature before shape compensation, the deformation of the surface in the normal direction becomes very uniform.

**Figure 12:**
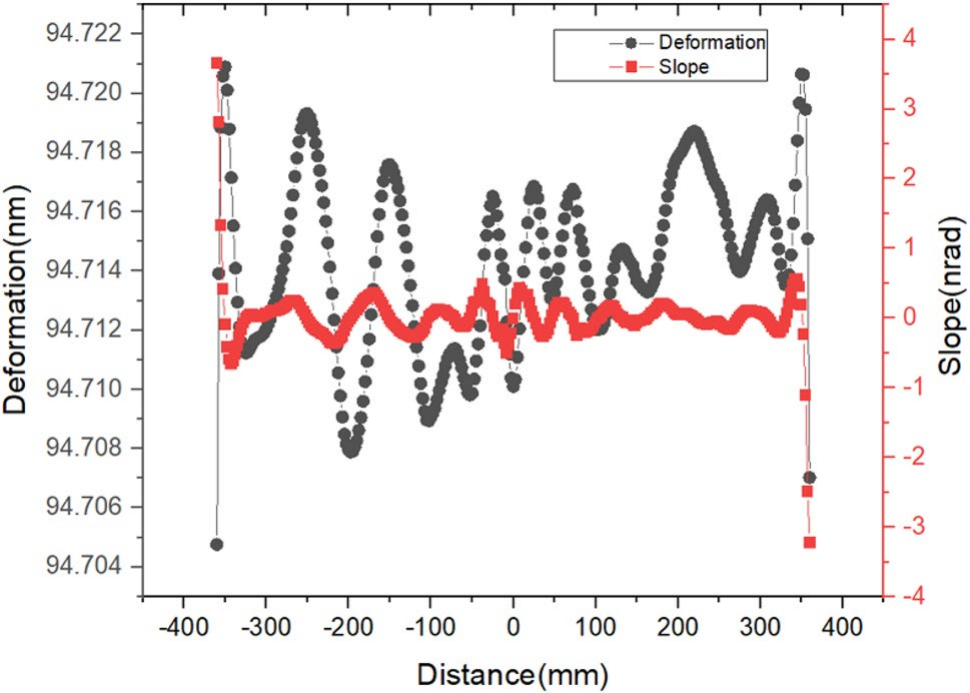
Deformation and slope curve of the centerline of footprint.

From Fig. 12, it can be seen that although the maximum deformation of the centerline in the meridional direction is over 94.7 nm within the range of [− 360 mm, 360 mm], the height error PV is only 16.1 pm, and the RMS value is 9.2 pm. In addition, the slope error PV is 6.8nrad, and the RMS value is 0.4nrad. Obviously, both the height error and slope error are much smaller than the requirements.

These results are obtained assuming that the mirror initial surface is perfect (without shape error) and that the effects caused by gravity and clamping can be ignored. In fact, the best mirrors currently produced by JTEC Corporation still have errors^22^. If the mirror initial deformation can be controlled within a certain range, the REAL scheme can be used to correct the mirror shape to meet the requirements. In the case, using the MHCKF model and optimization algorithm mentioned above, the height error caused by X-ray heat load can be reduced to an order of picometers.

**Figure 13:**
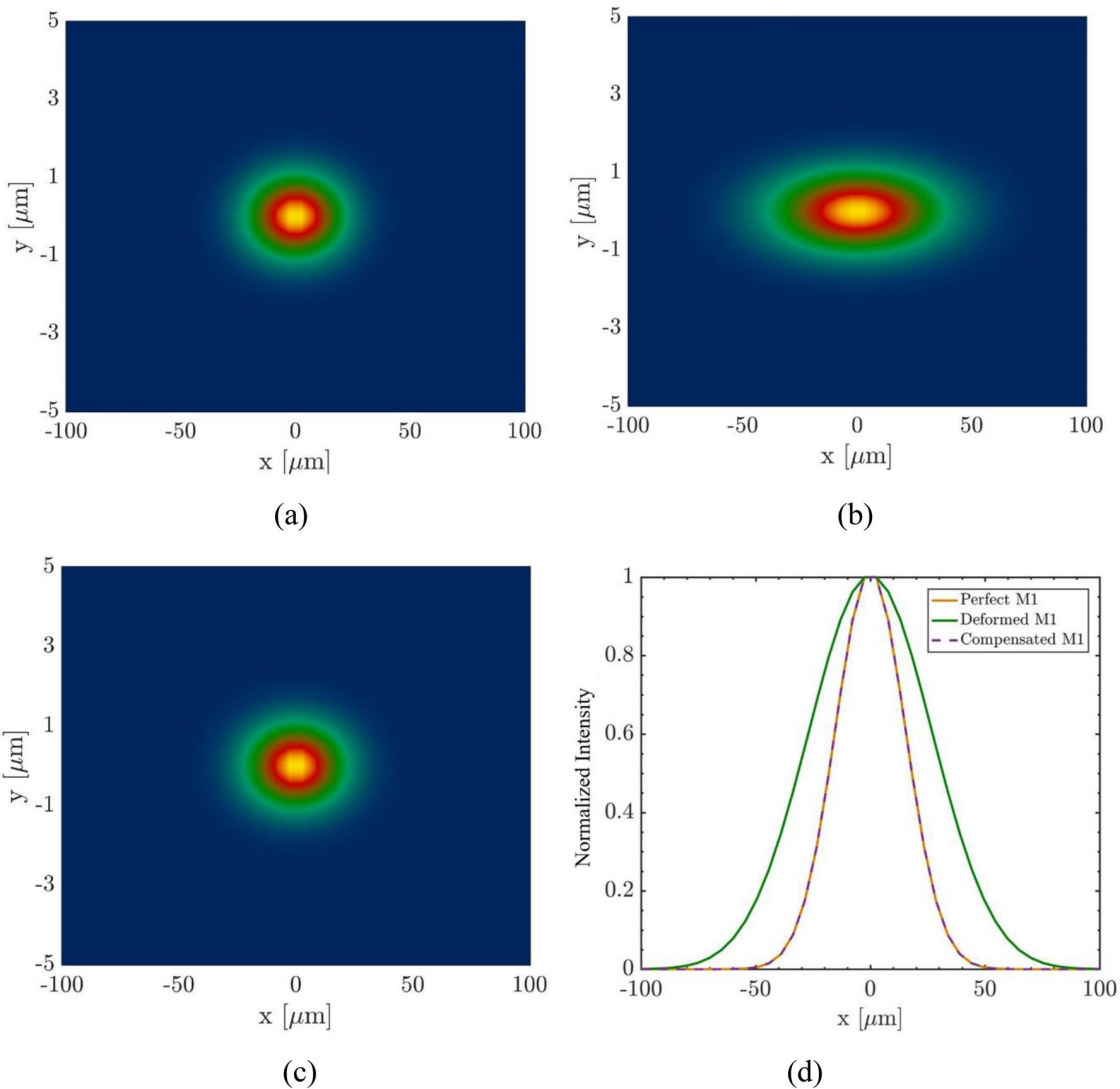
Wavefront Simulation for FEL beam passing through : (**a**) the perfect M1; (**b**) the M1 with thermal distortion; (**c**) the M1 after shape compensation; (**d**) intensity distributions.

To evaluate the effect of mirror shape on wavefront, wavefront simulations have been performed by means of Synchrotron Radiation Workshop (SRW) code^23^ before and after the shape compensation. The transverse beam profiles and horizontal intensity distributions are shown in Fig. 13 when the FEL beam passes through three different mirrors and reaches the horizontal focus. As can be seen, the wavefront after the mirror shape compensation is almost identical to the ideal wavefront.

**Figure 14:**
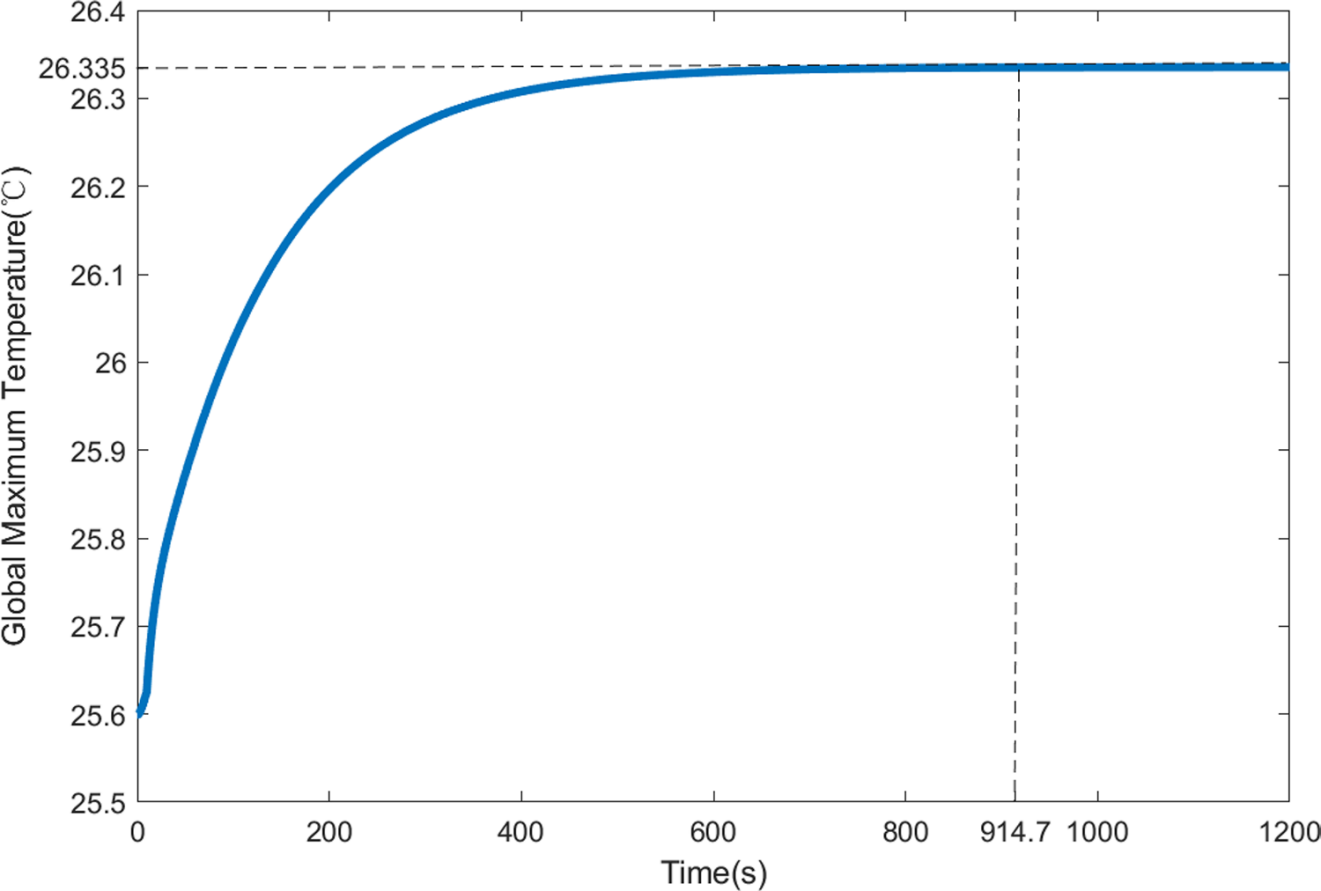
The curve of maximum temperature versus time.

The aforementioned finite element simulations used steady-state analysis to obtain the thermal equilibrium state. An evaluation of the time required for surface shape compensation was also performed. In other words, the initial state for optimization was taken as the equilibrium state before compensation (as shown in Fig. 4a), and timing started from the application of optimized heat fluxes. The maximum temperature of the entire system was used as an evaluation criterion until it reached thermal equilibrium (as shown in Fig. 11a). Utilizing the transient analysis of Ansys, the curve depicting the variation of the maximum temperature with time was obtained, as shown in Fig. 14. It can be observed that the maximum temperature gradually increased from an initial value of 25.6 °C and reached thermal equilibrium after approximately 914.7 s, which is comparable to the time required for balancing through PZT correction of the mirror shape^24^.

### ·The number of heaters

The length of the copper blade in Fig. 2 is 800 mm. In addition to the 30 mm-long heater introduced earlier, different heaters with lengths ranging from 35 to 55 mm and a step size of 5 mm are considered. These parameters are listed in Table 3.

**Table 3:** The length of each heater and Spacing between two heaters vs The number of heaters.

The length of each heater(mm)	Spacing between two heaters (mm)	The number of heaters
30	2	25
35	3	21
40	2	19
45	2	17
50	3	15
55	3	13

**Figure 15:**
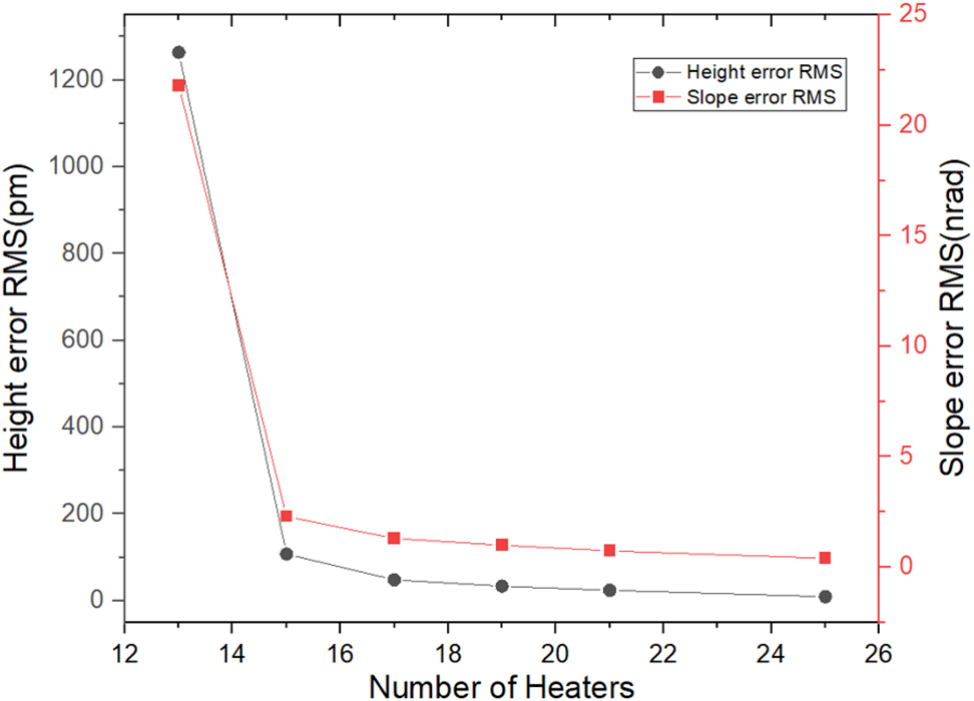
The number of heaters vs. Height error RMS and Slope error RMS.

To evaluate the influence of the number of heaters on shape errors, in addition to the aforementioned 25 heaters, the above optimization algorithm was also used to optimize other cases including 21, 19, 17, 15, and 13 heaters. From Fig. 15, it can be seen that except for the case of 13 heaters, both the height error and slope error are satisfied. For the case of 13 heaters, even if the height error is greater than 0.9 nm and does not meet the requirements, the slope error is still very small. In short, the more heaters there are, the better the final mirror shape will be. As long as the number of heaters is not less than 15, the compensated shape error will definitely meet the requirements.

In Table 4, a comparison is also made between the height error PV values obtained using the MHCKF model and those obtained by Ansys Workbench. It can be seen that all the results calculated by Ansys Workbench are smaller than those obtained by the MHCKF model. When the height error PV value is at the nanometer scale, the data obtained by the two methods are very close. The more heaters there are, the greater the difference between them.

**Table 4:** The height error PV calculated by MHCKF vs Ansys Workbench.

The number of heaters	The height error PV calculated by	
	MHCKF (nm)	Ansys Workbench(nm)
13	1.796	1.789
15	0.155	0.148
17	0.0757	0.0692
19	0.0541	0.0491
21	0.0427	0.0333
25	0.0456	0.0161

### Higher repetition rate

Although M1 is designed to operate at a repetition rate of up to 100 kHz, the case of operating at a higher repetition rate of 500 kHz is also considered. And the mirror will absorb five times more thermal power than initially. For the case of 15 heaters, the deformation and slope curves can be obtained when the shape error is minimized.

**Figure 16:**
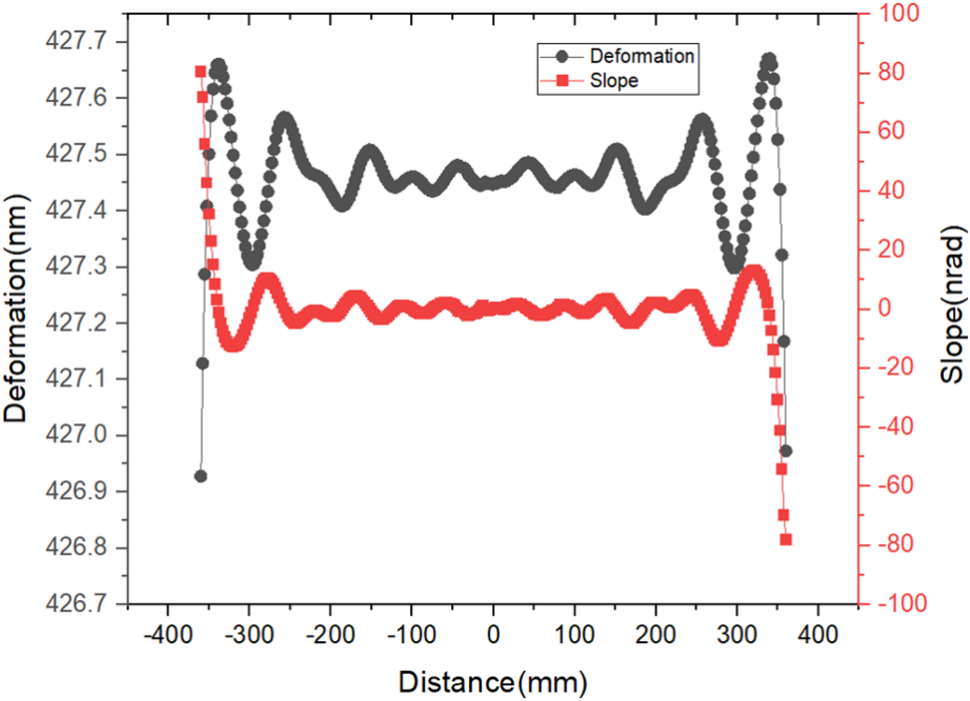
Deformation and slope curve of the central line of footprint.

As can be seen from Fig. 16, although the deformation of the centerline in the meridional direction within the footprint is more than 426.9 nm, the height error PV is only 0.74 nm and the RMS value is 0.54 nm. And the slope error PV is 158.5nrad and the RMS value is 11.9nrad. Therefore, for the case of 15 heaters and a 500 kHz repetition rate, the shape errors can meet the requirements, using the MHCKF model. Thus, with the mirror and shape compensation system unchanged, these results make it possible for the mirror to operate at a higher repetition rate if there is no material damage on the mirror surface.

### Film coefficient

**Figure 17:**
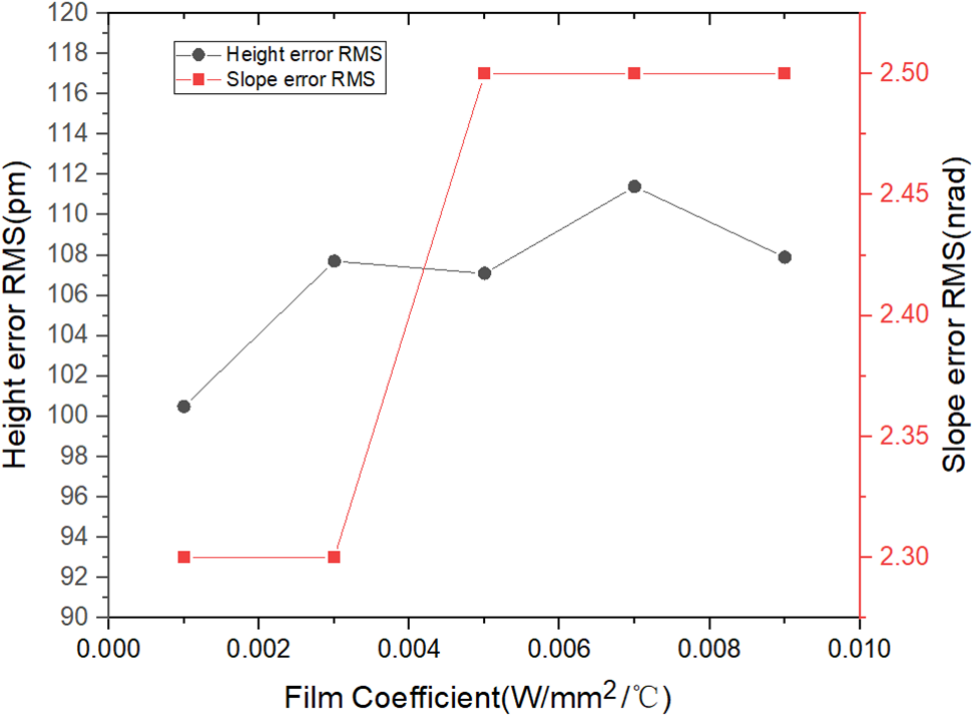
The Height error RMS and slope error RMS versus film coefficient.

In the model shown in Fig. 2, the thermal power from the X-ray and resistive heaters is carried away by cooling water flowing through copper tube. To study the influence of water flow rate on shape error, the effective film coefficients varied between 1E − 3 and 9E-3 (W/mm^2^/°C) with an interval of 2E-3 (W/mm^2^/°C) for the case of 15 heaters. The height and slope errors RMS were calculated using the optimization algorithm mentioned above. From Fig. 17, it can be seen that the difference between the maximum and minimum height errors RMS is only about 10 pm, and the difference in slope errors RMS is about 0.2 nrad. Therefore, in this case, the effect of the film coefficient on mirror shape error is small, and a low film coefficient (or low water flow rate) is sufficient.

### The length of copper tube

In the previous simulation analysis, both the copper blade and the copper tube had the same length. According to the paper from Xu and Zhang, the length of the copper tube has a certain influence on the mirror shape. Therefore, for the case of 15 heaters, an analysis was conducted to examine the effect on the shape errors when the length of the copper tube varied from 600 to 800 mm at intervals of 50 mm, while the length of the copper blade remained constant.

**Figure 18:**
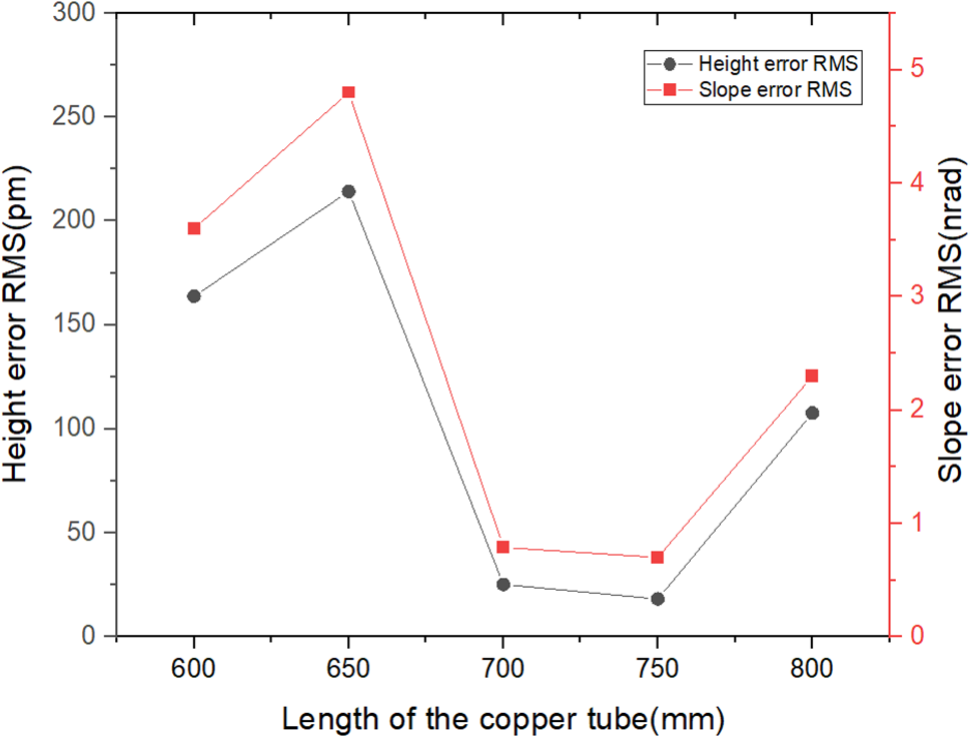
The Height error and slope error RMS versus the length of the copper tube.

As can be seen, as shown in Fig. 18, there is a change of nearly 200 pm between the maximum and minimum height error and a 4nrad difference between the maximum and minimum slope error. At a length of 750 mm, the height error and slope error are similar to those using the 21 resistive heaters in Fig. 15. So, the effect of the length of the copper tube on the mirror shape is verified again.

## Conclusion

This paper establishes MHCKF model for compensating the mirror surface shape using multiple resistive heaters and proposes an optimization algorithm. Based on them, an optimization design of heat fluxes applied on the copper blade in the FEL-1 beamline at S3FEL is carried out. From the shape error and wave-optics simulation, it can be seen that almost perfect wavefront propagation can be achieved after the mirror shape compensate. In addition, some factors affecting the shape error are analyzed, such as the number of heaters, higher repetition rate, film coefficient, and the length of the copper tube. It can be seen that the MHCKF model and optimization algorithm are very effective in quickly obtaining the heat fluxes generated by multiple heaters while obtaining the minimum shape error. This method can also be used for future real-time online shape control. The next step is to make a prototype and perform the necessary tests (Supplementary Information).

## Supplementary Information

Supplementary Information.

## Data Availability

The datasets generated during and/or analyzed during the current study are available from the corresponding author on reasonable request.
